# Supplementary material for: Interactions between climate change, urban infrastructure and mobility are driving dengue emergence in Vietnam
Source: Nat Commun. 2023 Dec 11;14:8179. doi: 10.1038/s41467-023-43954-0 (PMC10713571; doi:10.1038/s41467-023-43954-0)
Supplement: Supplementary file 3 — Reporting Summary [file 41467_2023_43954_MOESM3_ESM.pdf]

nature portfolio

Corresponding author(s): Rory Gibb
Last updated by author(s): 16/11/23

Reporting Summary

Nature Portfolio wishes to improve the reproducibility of the work that we publish. This form provides structure for consistency and transparency in reporting. For further information on Nature Portfolio policies, see our Editorial Policies and the Editorial Policy Checklist.

Statistics

For all statistical analyses, confirm that the following items are present in the figure legend, table legend, main text, or Methods section.

n/a	Confirmed	
☐	☒	The exact sample size (*n*) for each experimental group/condition, given as a discrete number and unit of measurement
☐	☒	A statement on whether measurements were taken from distinct samples or whether the same sample was measured repeatedly
☐	☒	The statistical test(s) used AND whether they are one- or two-sided<br>*Only common tests should be described solely by name; describe more complex techniques in the Methods section.*
☐	☒	A description of all covariates tested
☐	☒	A description of any assumptions or corrections, such as tests of normality and adjustment for multiple comparisons
☐	☒	A full description of the statistical parameters including central tendency (e.g. means) or other basic estimates (e.g. regression coefficient) AND variation (e.g. standard deviation) or associated estimates of uncertainty (e.g. confidence intervals)
☐	☒	For null hypothesis testing, the test statistic (e.g. *F*, *t*, *r*) with confidence intervals, effect sizes, degrees of freedom and *P* value noted<br>*Give P values as exact values whenever suitable.*
☐	☒	For Bayesian analysis, information on the choice of priors and Markov chain Monte Carlo settings
☒	☐	For hierarchical and complex designs, identification of the appropriate level for tests and full reporting of outcomes
☐	☒	Estimates of effect sizes (e.g. Cohen's *d*, Pearson's *r*), indicating how they were calculated

Our web collection on statistics for biologists contains articles on many of the points above.

Software and code

Policy information about availability of computer code

Data collection	The epidemiological data underpinning the study were collected over 23 years of national dengue surveillance by Vietnamese national heath institutes, and were processed using R pipelines to produce a standardised format aligned with geographical metadata. The Earth observation and climate model data used to generate covariates were collated from a variety of sources listed in the paper with a detailed description of processing provided in Methods and Supp. Text 1, including the R packages used in this processing. All analyses were carried out using the R packages dplyr (v1.1.0), magrittr (v2.0.3), raster (v3.6.21), sf (v1.0.7), exactextractr (v0.8.2) and INLA (v21.7.10.1).
Data analysis	Spatial visualisation and analysis was conducted using the R packages 'sf' and 'raster'. Spatiotemporal regression models were developed and fitted within the 'INLA' package in R (v21.7.10.1). Custom modelling scripts were developed for block cross-validation experiments and designed to run on an institutional HPC cluster (University College London's Myriad system). All code is provided within the accompanying repository, which contains a subset of the dengue epi data sufficient to reproduce the models and demonstrate script functionality. These are provided at the following Zenodo DOI link: http://dx.doi.org/10.5281/zenodo.10159288

For manuscripts utilizing custom algorithms or software that are central to the research but not yet described in published literature, software must be made available to editors and reviewers. We strongly encourage code deposition in a community repository (e.g. GitHub). See the Nature Portfolio guidelines for submitting code & software for further information.

Data

Policy information about availability of data

All manuscripts must include a data availability statement. This statement should provide the following information, where applicable:

- Accession codes, unique identifiers, or web links for publicly available datasets
- A description of any restrictions on data availability
- For clinical datasets or third party data, please ensure that the statement adheres to our policy

Dengue surveillance data for a subset of 4 Vietnamese provinces (Dak Lak, Khanh Hoa, Ha Noi and Dong Nai) are shared in the study repository to demonstrate analysis pipeline functionality. The nationwide dengue incidence data underlying these results are the property of the Vietnam Ministry of Health and are available from Phan Trong Lan, General Department of Preventive Medicine, MOH (phantronglan@gmail.com). All other data used in analyses were accessed from open sources. Land cover data was accessed from ESA-CCI (https://www.esa-landcover-cci.org/), census-based population, sanitation, housing and mobility data from the Vietnam General Statistics Office (https://www.gso.gov.vn/en/homepage/) and climate reanalysis data from Copernicus (ERA5-Land https://cds.climate.copernicus.eu/cdsapp#!/dataset/reanalysis-era5-land; WFDE5 https://cds.climate.copernicus.eu/cdsapp#!/dataset/derived-near-surface-meteorological-variables). Processed versions of these datasets used in analyses are provided in the study repository http://dx.doi.org/10.5281/zenodo.10159288

Research involving human participants, their data, or biological material

Policy information about studies with human participants or human data. See also policy information about sex, gender (identity/presentation), and sexual orientation and race, ethnicity and racism.

Reporting on sex and gender	N/A
Reporting on race, ethnicity, or other socially relevant groupings	N/A
Population characteristics	N/A
Recruitment	N/A
Ethics oversight	N/A

Note that full information on the approval of the study protocol must also be provided in the manuscript.

Field-specific reporting

Please select the one below that is the best fit for your research. If you are not sure, read the appropriate sections before making your selection.

☐ Life sciences ☒ Behavioural & social sciences ☐ Ecological, evolutionary & environmental sciences

For a reference copy of the document with all sections, see nature.com/documents/nr-reporting-summary-flat.pdf

Behavioural & social sciences study design

All studies must disclose on these points even when the disclosure is negative.

Study description	The study is a quantitative, spatiotemporal modelling analysis carried out on 23-years of dengue surveillance panel data from Vietnam, using monthly administrative-level case counts for 667 districts. We fitted spatiotemporal statistical models (Bayesian inference framework) to the full dataset to infer relationships between dengue incidence and key climatic and socio-environmental covariates, used block cross-validation experiments to understand covariate importance, and projected the long-term effects of temperature change on dengue incidence across Vietnam using historical reanalysis data.
Research sample	Rather than a sample, our study used a full dataset of dengue case surveillance collected from across the entire study period from Vietnam in order to provide a detailed view of trends in dengue disease over space and time. 174,936 monthly dengue fever case counts from 667 districts between May 1998 and April 2021. Observations were aggregated surveillance-based case counts at the administrative unit level so contained no individually identifiable information.
Sampling strategy	We used a complete panel time series from across the study period (monthly case counts per district across the full historical time-series from 1998 to 2021). These were not sampled or subsampled.
Data collection	Dengue case counts were collected through hospital-based passive surveillance using the national dengue case definition and were based on clinically diagnosed (suspected) cases. Individual cases were diagnosed by clinicians at hospital level, then counts were aggregated and reported at administrative-2 level (district) through the national dengue surveillance system.
Timing	May 1998 to April 2021.

Data exclusions	No data were excluded.
Non-participation	No participants were involved in the study as the data were aggregated from administrative-level disease surveillance system.
Randomization	Data were not collected in an experimental design and instead were administrative level disease cases collected during surveillance activities, so randomization was not applicable.

Reporting for specific materials, systems and methods

We require information from authors about some types of materials, experimental systems and methods used in many studies. Here, indicate whether each material, system or method listed is relevant to your study. If you are not sure if a list item applies to your research, read the appropriate section before selecting a response.

Materials & experimental systems

n/a	Involved in the study	
☒	☐	Antibodies
☒	☐	Eukaryotic cell lines
☒	☐	Palaeontology and archaeology
☒	☐	Animals and other organisms
☒	☐	Clinical data
☒	☐	Dual use research of concern
☒	☐	Plants

Methods

n/a	Involved in the study	
☒	☐	ChIP-seq
☒	☐	Flow cytometry
☒	☐	MRI-based neuroimaging
